# Supplementary material for: Phase locking and multistability in the topological Kuramoto model on cell complexes
Source: Nat Commun. 2026 Jul 25;17:7409. doi: 10.1038/s41467-026-75565-w (PMC13408971; doi:10.1038/s41467-026-75565-w)
Supplement: Supplementary file 1 — Transparent Peer Review file [file 41467_2026_75565_MOESM1_ESM.pdf]

nature portfolio

Peer Review File

Phase locking and multistability in the topological Kuramoto model on cell complexes

Corresponding Author: Dr Iva Bačić

This file contains all reviewer reports in order by version, followed by all author rebuttals in order by version.

Version 0:

Reviewer comments:

Reviewer #1

(Remarks to the Author)
In this paper, the authors find theoretical conditions for twisted states and multistability for the Topological Kuramoto model on cell and simplicial complexes. They apply tools from algebraic topology and theory of higher-order networks to find exact conditions for phase locking states with given winding numbers and study their stability. They show examples of different cell and simplicial complexes.

Overall, this paper presents interesting and innovative results. The developed theory is elegant and clever, from the application of a nonlinear topological Kirchhoff law, to the exploitation of the kernels of the boundary operators to build some auxiliary variables. However, the resulting paper is extremely heavy and difficult to read, making it accessible only to a very narrow audience, in contrast to the broad aim of Nature Communications. The reader is not helped at all, most quantities are not defined and the understanding of even the basic concepts relies on previous knowledge of some other papers, which are already themselves very difficult to read and known in details by a restricted audience.

Given all the above, although we value the results and the theory developed, we do not think that the paper can be accepted in this form and needs some major revisions, not in terms of the results, but rather in terms of the presentation. More background needs to be given, as well as intuition of what the authors are doing and why. Also, some explicit examples and actual time series of twisted states of the Topological Kuramoto would also be of great help, including some videos.

Below we list some detailed comments:

_The Introduction is well written. However, given that the Results part is extremely heavy and difficult to read, at least some more details and highlights should be given in the Introduction.

_In Fig. 1, while it is clear how to go from Step 1 to Step 2, the last passage is not clear.

_The boundary and co-boundary operators are not defined. Besides the definition, at least an explicit example should be given. The github is very nice, but some details should be given in the manuscript.

_Something more about the Hodge decomposition should be said. Also, explicitly mentioning the connections between the boundary and co-boundary operators with the discrete divergence and gradient could help the reader understand what the authors are doing.

_The difference between cell and simplicial complexes is not explicitly made. These are not yet concepts mastered by a broad audience.

_Fig. 2 is the only Figure that is completely clear and easy to understand. Nonetheless, it is not mentioned explicitly that the orientation is needed to have a base for the boundary operators, while, even to authors familiar with network science, this may look like a directionality.

_It would greatly help if the structures of Fig. 2 would be used to explicitly compute the conditions for phase locking.

_Not all readers may be familiar with the Topological Kuramoto. A brief recap and an explanation of what it means to project a cochain one dimension up or one dimension down should be given. Also, given that these are the variables that are then studied, some discussion of the dynamics of the latter could help contextualize the results.

_Some more details behind the rationale of the topological Kirchhoff condition could be given to guide the reader on what the authors are doing and why.

_At least an explicit example of the matrices C should be given. Also, some more details on the kernels of the boundary operators and their relation with the kernel of the Hodge Laplacian and the Betti numbers could be given, so to have more intuition of what these quantities mean topologically.

_The division in partitions where the cosine is positive or negative is clever. But can the authors give more intuition of what it means? Again, an explicit example would help.

_The literature on the topic is not much and already a bit difficult. Using more standard terms, like n-simplices, n-chains, n-cells, n-cochains would help. Even with maybe a small dictionary at the beginning, so that every reader is on the same page. In fact, the notation is often inconsistent (e.g., sometimes "nodes", some others "vertices").

_Can the authors explain in details what are the conditions of Eq.(22)? This is what is then plot to show the main results, so it is important to have an intuition of what it means. Same goes for the later introduced variable \zeta.

_In Figs. 3 and 4 (as well as the others), it is very difficult to distinguish between filled and empty circles. In general, all Figures are extremely difficult to understand, and the captions do not help much in grasping what it is presented.

_The lower panel in Fig. 4 is not clear.

_It is interesting how heterogeneous frequencies can be used to control the emergence of multistability. Could the authors make an explicit example?

_The authors claim that cell complexes support richer multistability than simplicial complexes of the same dimension. First of all, we are not entirely sure about this, as from Fig. 6 it seems that the cube (cell complex) has the same number of stable state than the octahedron (simplicial complex), and the dodecahedron (cell complex) the same as the icosahedron (simplicial complex). If we are wrong and did not understand correctly, can the authors explain the Figure? Assuming that their claim is correct: (1) is it valid for any n-cochain? (2) how is it compatible with the fact that, for 1-cochains, synchronization is obstructed on simplicial complexes while not on cell complexes? (so, one would assume a richer dynamics where there cannot be synchronization).

_The authors seem to use "simplex" and "simplicial complex" to mean the same thing, but this causes confusion. A simplex is just one part of the structure, e.g., a 0-simplex is a node, a 1-simplex is a link, etc., while the structure is a simplicial complex. This should be fixed. With such definitions, Fig. 8 results extremely confusing.

Minor comments:

_line 320: does not corresponds;
_i.e. should be followed by a comma.

(Remarks on code availability)

Reviewer #2

(Remarks to the Author)
I co-reviewed this manuscript with one of the reviewers who provided the listed reports. This is part of the Nature Communications initiative to facilitate training in peer review and to provide appropriate recognition for Early Career Researchers who co-review manuscripts.

(Remarks on code availability)

Reviewer #3

(Remarks to the Author)
The authors consider a topological Kuramoto model generalized to cell complexes. They present a method for identifying all phase-locked states and their stability. They systematically applied their method to structures of increasing dimensions, and show that multistability requires a minimum number of boundary cells in the structure, and that multistability can be reduced by introducing asymmetry in the natural frequencies.

I found the results very interesting, novel, and strong. The text is well written, although a bit on the technical side, and I appreciate the effort in making the figures. I believe the method to identify the phase-locked states, and the results on multistability, will have an impact on future research in the area. Below, I mainly detail what was less clear to me, with suggestions to further improve the clarity and interpretation of some of the key mechanisms/variables, which whill hopefully help the manuscript have the impact I expect it will.

MAJOR COMMENTS

- The text is technical, which is not a problem, because it shows strong analytical results. However some key aspects could be clarified:
. l162: define what phase locked states are in the context of the topological kuramoto (and how they differ from the standard Kuramoto). They are central to the paper but not defined.
. \zeta is the key control parameter in the main figures (figs 3, 4, 6, 7), but is only introduced as "vectors of free coefficients which parametrize the homogeneous solution space". Add a "physical" interpretation of their origin and of what they control.
. l 341 and 358 and throught: w=0 and w!=0 are described as symmetric and asymmetric. Please explain why. w is used as a control parameter in key results (eg figs 4, 7) and it should be clear.
. Key results and figures discuss different possible partitions. It was not fully clear to me what those different partitions represent ("physically"). I initially thought was unique and defined based on the sign of cos(theta).
- z is introduced as " a vector of integers" and "in the case of ordinary graphs, as winding vectors" (l 263). This interpretation is never used in the description of the results also z is often explicitly given in many cases of "Class 3: simplexes". At some point I even found myself wondering if z = (0, 0, 0) was corresponding to full sync (which it does not). So remind the readers and help them interpret those values.
In the discussion, consider also adding some interpretations to the results based on the above.

- l 549: " we showed that multistability emerges when boundaries contain more than four cells". This is very interesting. Do you have any idea of where this number 4 comes from? Why 4?

- Where relevant, compare your results with the literature on hypergraphs.

MINOR COMMENTS

- l49: "revealing universal features absent in conventional networks, such as abrupt synchronization". This sounds mistaken, abrupt synchronization has been shown also in non topological networks.

- l90-112: Cell complexes are not common as simplicial complexes or hypergraphs in synchronization literature and need to be introduced with more precision or care. There is not explicit definition of what a cell complex is. Maybe also add a comparison with simplcial complexes and hypergraphs too where possible.

- l97: "but many real-world systems are better described by cell complexes" add a reference and examples, and clarify why they are better described by cell complexes

- l229: is there a reason/name why \psi_{sp} is called "sp"?

- l249: I only understood later (first example) that the notation full/empty circle based on the cos(theta) sign was because that sign determines stable/unstable (Eq 11). Maybe remind it there.

- l258-260: "The question whether a solution candidate (16) exists (...) can thus be formulated as [Eq 21]." is potentially confusing. If Eq 21 is satisfied, I'm guessing that it means that a "a candidate solution (16) can be expressed as (12)", and not the opposite (the phrasing does not specify which holds). If so, rephrase accordingly to make that explicit.

- l268-281: "To summarize, we propose the following algorithm" it's not immediately clear that 4 points below are a summary of what was described before. "2. Choose a partition" but I thought the partition was unique and defined based on the sign of cos(theta)? Point 4: When is it not necessary to the check the stability from the Jacobian?

- l337: "the of the" missing word, probably kernel?

- l330: "solutions candidates" -> "solution candidates" or maybe even better "candidate solutions" throughout

- Fig 3: The caption should state what the x- and y- variables are (and what they represent), even if state in the main text.

- Fig 8: Would be most naturally displayed as a table (in text, rather than as an figure/image)

- me being picky: text sub/superscripts should generally not be in italic/math mode. Use {\rm down} instead of {down} for example.

(Remarks on code availability)

Reviewer #4

(Remarks to the Author)
Potentially Important steps on extending the analysis of topological Kuramoto models on simplicial complexes to cell complexes.

I think the image in Figure 1 is confusing and possibly inaccuarate whe interpreted in a natural way. If the symbols \psi_i are called phase differences (as the caption says), then it should be their algebraic sum that equals 2z\pi -- not their arcsin.

I am also confused about the use of the arcsin function. I assume the document means the principal branch in [-pi/2,pi/2], but are counterclockwise angular difference always guaranteed to take value in that interval?

The presentation/results on phase locking is very technical and hard to follow. There are many variables (typically without an intuitive name), many intermediate steps, no example cell complex and corresponding Kuramoto, and overall limited insight into the big picture.

suggestions:

1. give names to key critical variables, e.g., the B matrices, the angles \theta^[+] and \theta^[-],

2. prepare a low dimentional example showing the most important steps, possibly to be included in a figure or in the supplementary information. (e.g., what are the B matrices in Figure 2? what are the decoupled equations (5) and (6) for the two examples in Figure 2?)

Overall, the main contribution is Algorithm 1, which identifies all distinct phase-synchronized states in cell complexes and their stability. I am supportive of this result: I would appreciate (i) a discussion about number of equilibrium points and computational complexity, (ii) a discussion about the history and available software implementations of Algorithm 1 for standard Kuramoto models and Kuramoto models on simplicial complexes, and (iii) invariance under Abelian action (rotation of all angles by a constant angle) which is hidden in the current treatment.

Beside my concerns about the clarity and accuracy of the presentation, I have to say that the existence of Algorithm 1 is relatively natural (given similar results for the standard Kuramoto) and I am slightly unsure about whether this result deserves to appear in Nature Communication.

(Remarks on code availability)
I did not review the code

Version 1:

Reviewer comments:

Reviewer #1

(Remarks to the Author)
The authors did an excellent job of revising the paper. The new version is clearer, and the overall presentation has significantly improved. While some aspects still require refinement, this version is already of very good quality.

The Kirchhoff conditions are now intuitively clear, even though a few steps in the computation remain somewhat difficult to follow (see below). The examples are highly illustrative and effective. I particularly appreciated Figs. 3 and 4 along with their respective captions. Having read this revised version, I can now appreciate the beautiful theory developed by the authors.

Given that this is paper is considered for publication in a broad-audience journal, I believe the authors should endeavor to make their topic even more reader-friendly. They should avoid taking foundational concepts for granted when introducing topological signals and cell complexes. Moreover, a very brief overview of the dynamics of topological signals, beyond just the Topological Kuramoto model, would greatly benefit the community and familiarize more readers with this emerging field.

Specific comments and suggestions are as follows:

- At the beginning of Results, I think it would be best to write "networks" instead of "graph" in the title of the section. In fact, right in the first sentence the authors use "network".

- In general, throughout the manuscript, both "graph" and "networks" are found. I'd suggest the use of one of the two, mentioning that they are equivalent at the beginning (given the community where this paper will circulate, I'd suggest the use of "network").

- After Eq. 3, I think it would be more correct to write "is the graph (network) Laplacian", rather than "is a graph Laplacian". If the authors write "a", it should be explained that this is the combinatorial Laplacian, not to be confused with the consensus nor the random walk one.

- Fig 1 is improved, but I still think it's not too clear, especially for scholars coming from networks and not too familiar with higher-order interactions. First of all, it's not clear that edges are not directed but it's just an orientation needed to define the boundary operators. Then, the faces are not well displayed. The cell-complexes both look like directed networks with some kind of vector field in the faces. I think this figure needs to be further improved (especially panels a and b).

- It should be noted somewhere that, while the Laplacian is uniquely defined, the boundary operators are not and in principle other orientations would be possible.

- Given the broad audience of this journal, some more explanations on L_up and L_down should be given.

- I am not sure the dimensions of the cell-complexes are correct. If the cube has really n=3, then there should also be a volume-face, which, if I understand correctly, is not present. So n=2 for both cell complexes.

- When introducing n-cochains, rather than "vectors defined on n-cells", I think it clearer to write "state variables defined on n-cells". Also, an analogy with differential forms could help readers not familiar with this.

- When talking about the n-th Betti number, it should be specified that it is the number of n-dimension cycles (maybe also some examples could be given). In the present phrasing, it seems that the cycles can be of any dimension.

- In Fig 2, it should be mentioned what are z^{[+/-]} and possible given an explicit example. Also, an explicit mention of what is the winding number in the case of network should be given, as not every reader may be familiar with it.

- The authors can now get an intuition on what the matrices C are, but still it's not completely clear to me how they can be obtained. Is there an exact relation with the corresponding boundary matrices which would satisfy the same condition?

- When obtaining the Kirchhoff conditions, I could follow up until before Eq. 19, then I got lost. Why is the general solution written as in Eq. 19?

- After Eq. 21, the authors say that the frequency vector can be used as a control parameter. But the frequency can be rescaled with the coupling strength, so it should be specified that the coupling strength needs to be fixed. Or, alternatively, that one of the two can be used as a control parameter.

- The use of "symmetric" and "asymmetric" in dynamics on networks is tricky, because the reader might think that the support is somehow asymmetric. I suggest finding an alternative or specify, e.g., "permutation-symmetric", etc.

- The fact that heterogeneous frequencies can turn unstable locked states into stable ones is very interesting. Is there a pairwise analogous of this?

- In the section discussing simplicial complexes, there's a typo when introducing Table I in the text ("I" is missing).

(Remarks on code availability)

Reviewer #2

(Remarks to the Author)
I co-reviewed this manuscript with one of the reviewers who provided the listed reports. This is part of the Nature Communications initiative to facilitate training in peer review and to provide appropriate recognition for Early Career Researchers who co-review manuscripts.

(Remarks on code availability)

Reviewer #3

(Remarks to the Author)
The authors have addressed all my concerns. I thank them for their clear responses and careful revision.

(Remarks on code availability)
The readme clearly details the structure of the code repo and the system requirements.

Reviewer #4

(Remarks to the Author)
The paper has been substantially reviewed. So much so that it appears to be a completely new document. I approve of the extensive changes. Each of my comments during the previous revision has been satisfactorily addressed and, even more, the revision is clearly compelling. The positive parts of the original submission has been strenghtened and, as such, I am glad to recommend acceptance.

(Remarks on code availability)
n/a

Open Access This Peer Review File is licensed under a Creative Commons Attribution 4.0 International License, which permits use, sharing, adaptation, distribution and reproduction in any medium or format, as long as you give appropriate credit to the original author(s) and the source, provide a link to the Creative Commons license, and indicate if changes were made.
In cases where reviewers are anonymous, credit should be given to 'Anonymous Referee' and the source.
The images or other third party material in this Peer Review File are included in the article's Creative Commons license, unless indicated otherwise in a credit line to the material. If material is not included in the article's Creative Commons license and your intended use is not permitted by statutory regulation or exceeds the permitted use, you will need to obtain permission directly from the copyright holder.
To view a copy of this license, visit https://creativecommons.org/licenses/by/4.0/

REPLIES TO THE REVIEWER COMMENTS.

First, we thank the reviewers for their thorough reading of our work, and their positive and constructive feedback. In the following, we provide a detailed response to the reports. Original comments are given in black italic font, The replies to the reviewers are given in blue (Our reply: sample comment), while changes to the manuscript are given in purple (Changes to the manuscript: sample text).

REVIEWER #1

In this paper, the authors find theoretical conditions for twisted states and multistability for the Topological Kuramoto model on cell and simplicial complexes. They apply tools from algebraic topology and theory of higher-order networks to find exact conditions for phase locking states with given winding numbers and study their stability. They show examples of different cell and simplicial complexes.

Overall, this paper presents interesting and innovative results. The developed theory is elegant and clever, from the application of a nonlinear topological Kirchhoff law, to the exploitation of the kernels of the boundary operators to build some auxiliary variables. However, the resulting paper is extremely heavy and difficult to read, making it accessible only to a very narrow audience, in contrast to the broad aim of Nature Communications. The reader is not helped at all, most quantities are not defined and the understanding of even the basic concepts relies on previous knowledge of some other papers, which are already themselves very difficult to read and known in details by a restricted audience.

Given all the above, although we value the results and the theory developed, we do not think that the paper can be accepted in this form and needs some major revisions, not in terms of the results, but rather in terms of the presentation. More background needs to be given, as well as intuition of what the authors are doing and why. Also, some explicit examples and actual time series of twisted states of the Topological Kuramoto would also be of great help, including some videos.

Our reply: We thank the reviewer for the careful reading of our manuscript and for the positive assessment of the novelty and elegance of our results. We appreciate in particular the recognition of the theoretical framework based on the topological nonlinear Kirchhoff conditions.

We fully agree that the original presentation was too dense and not sufficiently accessible for a broad audience. Improving clarity, providing more intuition, and making the paper more self-contained have therefore been central goals of our revision.

Changes to the manuscript:

- We have restructured the beginning of the Results section to separate intuition from technical developments. The new section “Synchronization on graphs and cell complexes” introduces the dynamical systems perspective and provides an intuitive entry point, while the subsequent section “Properties of cell complexes” presents the required mathematical concepts with additional explanations and physical interpretation.
- We have substantially revised Fig. 1 and Fig. 2 and flipped the order of occurrence to better illustrate and motivate the key ideas and the overall approach.
- We have added time series of representative phase-locked states to Fig. 1 and Fig. 3 to provide a more direct dynamical illustration of the results.
- We now include a detailed derivation of all twisted states for the ring network as an explicit example, illustrating the general method step by step.

Below we list some detailed comments:

The Introduction is well written. However, given that the Results part is extremely heavy and difficult to read, at least some more details and highlights should be given in the Introduction.

Our reply: We thank the reviewer for this helpful suggestion. We agree that the Introduction should provide more guidance and intuition to prepare the reader for the technical developments in the Results section. We have therefore revised and extended the Introduction accordingly, with the aim of highlighting the key ideas and improving accessibility, and we have added examples throughout the manuscript.

Changes to the manuscript:

- We have extended the Introduction to convey the basic idea underlying the topological nonlinear Kirchhoff conditions.
- We have redesigned and repositioned Fig. 1 (former Fig. 2) to appear at the beginning of the manuscript. It now provides a visual overview of representative cell complexes, their boundary operators, and the associated dynamics.
- Throughout the manuscript, and in particular in the Introduction and early Results sections, we have added additional physical intuition to clarify the mathematical constructions and guide the reader through the main concepts.

In Fig. 1, while it is clear how to go from Step 1 to Step 2, the last passage is not clear.

Our reply: We thank the reviewer for pointing out this lack of clarity. We agree that the transition from the intermediate step to the final formulation was not sufficiently explained in the original figure.

To address this issue, we have redesigned the conceptual figure (now Fig. 2 in the revised manuscript) to explicitly illustrate all steps of the construction using a basic example (the tetrahedron). In addition, we have revised the corresponding text to better guide the reader through this transition.

Changes to the manuscript:

- We have redesigned the conceptual figure (now Fig. 2) and changed the order of the figures to improve the logical presentation. The new figure illustrates the key steps of the construction for a basic example and clarifies the final transition. It explicitly highlights the role of nodes, edges, and faces, and shows the corresponding variables $\psi_i^{[\pm]}$.
- We have rewritten the section introducing the topological nonlinear Kirchhoff conditions to closely follow Fig. 2, first explaining the general idea and then providing the mathematical formulation.

The boundary and coboundary operators are not defined. Besides the definition, at least an explicit example should be given. The github is very nice, but some details should be given in the manuscript.

Our reply: We thank the reviewer for this important comment. We fully agree that the definition and illustration of the boundary and coboundary operators were insufficient in the original manuscript. We have therefore revised this part of the paper to provide both a clear definition and explicit examples.

Changes to the manuscript:

- We have revised and extended Fig. 1 (Fig. 2 in the previous version), which now presents two explicit examples, the hexagonal ring and the cube. For both cases, we show the corresponding boundary operators alongside the structures, together with representative time series illustrating multistability.
- We introduce the boundary and coboundary operators in the section "Synchronization on graphs and cell complexes". Here, we omit technical details and work out the analogy to standard graph theory: "The topology of a cell complex is encoded by boundary operators. The operator $\boldsymbol{B}_{[n]}$ encodes which $(n-1)$-cells lie in the boundary of each n-cell. Thus, in the topological Kuramoto model, $\boldsymbol{B}_{[n]}$ describes interactions through lower-dimensional cells, while the coboundary operator $\boldsymbol{B}_{[n+1]}^{\top}$ describes interactions through higher-dimensional cells. For $n=1$, $\boldsymbol{B}_{[1]}$ is the usual node-edge incidence matrix and $\boldsymbol{B}_{[2]}$ is the edge-face incidence matrix (Fig. 1**c,d**)."

- We provide further explanations and technical detail in the section on cell complexes: "Boundary operators $\boldsymbol{B}_{[n]} \in \mathbb{R}^{N_{[n-1]} \times N_{[n]}}$ map n-cells to their $(n-1)$-dimensional boundaries. After fixing an orientation, their entries take values in $\{-1, 0, +1\}$, indicating whether a given $(n-1)$-cell appears in the boundary with positive or negative orientation, or not at all. The choice of orientation determines the signs in $\boldsymbol{B}_{[n]}$, but does not imply any physical directionality of the interactions. The transpose $\boldsymbol{B}_{[n]}^{\top}$ is called the coboundary operator. A fundamental property is that the boundary of a boundary vanishes, expressed by Eq. (6)."
- We further illustrate these concepts through the detailed example of the hexagonal ring.

Something more about the Hodge decomposition should be said. Also, explicitly mentioning the connections between the boundary and coboundary operators with the discrete divergence and gradient could help the reader understand what the authors are doing.

Our reply: We thank the reviewer for this helpful suggestion. We agree that providing additional context on the Hodge decomposition and its relation to discrete differential operators improves both clarity and intuition. We have therefore revised the corresponding paragraph to make these connections explicit.

Changes to the manuscript:

- We have added the following sentence to clarify the interpretation of the operators: "The operators $\boldsymbol{B}_{[n]}$ and $\boldsymbol{B}_{[n]}^{\top}$ can be interpreted as discrete differential operators. In particular, $\boldsymbol{B}_{[n]}$ acts as a discrete divergence, while $\boldsymbol{B}_{[n]}^{\top}$ acts as a discrete gradient. This interpretation becomes explicit for $n = 1$, where $\boldsymbol{B}_{[1]}$ reduces to the node-edge incidence matrix from standard graph theory. Each column of $\boldsymbol{B}_{[1]}$ contains one $+1$ and one -1, corresponding to the head and tail of an edge. Thus, $\boldsymbol{B}_{[1]}^{\top}\boldsymbol{u}$ assigns to each edge the difference of node values, i.e., a discrete gradient. Analogously, $\boldsymbol{B}_{[2]}^{\top}$ captures circulation around faces and can be viewed as a discrete curl."
- We have revised the discussion of the Hodge decomposition to provide a clearer interpretation of its components. The paragraph now reads: "The relation $\boldsymbol{B}_{[n]}\boldsymbol{B}_{[n+1]} = 0$ leads to the Hodge decomposition [1], a discrete analogue of the Helmholtz decomposition from vector calculus. Any vector $\boldsymbol{x}_{[n]} \in \mathbb{R}^{N_{[n]}}$ can be uniquely decomposed as

$$\boldsymbol{x}_{[n]} = \boldsymbol{x}_{[n]}^{H} + \boldsymbol{B}_{[n]}^{\top}\boldsymbol{z}_{[n-1]} + \boldsymbol{B}_{[n+1]}\boldsymbol{z}_{[n+1]}. \tag{1}$$

The second term is a gradient-like component induced from lower dimensions, while the third is a curl-like component describing circulation around higher-dimensional cells. In addition, there is a harmonic component $\boldsymbol{x}_{[n]}^{H}$, which satisfies

$$\boldsymbol{B}_{[n]}\boldsymbol{x}_{[n]}^{H} = \boldsymbol{0}, \qquad \boldsymbol{B}_{[n+1]}^{\top}\boldsymbol{x}_{[n]}^{H} = \boldsymbol{0}.$$

Thus, it lies in the intersection of the kernels of $\boldsymbol{B}_{[n]}$ and $\boldsymbol{B}_{[n+1]}^{\top}$, and its dimension is given by the nth Betti number, i.e., the number of independent cycles (holes) in the complex. It corresponds to conserved, divergence-free flows that circulate along topological cycles without being generated by local gradients or curls, and persist due to the presence of holes or loops."

The difference between cell and simplicial complexes is not explicitly made. These are not yet concepts mastered by a broad audience.

Our reply: We thank the reviewer for this important remark. We agree that the distinction between simplicial and cell complexes was not sufficiently explained in the original manuscript. We have therefore revised the Introduction and the section "Properties of cell complexes" to make this distinction explicit and more accessible.

Changes to the manuscript:

- We have extended the Introduction to explicitly introduce simplicial complexes as a special case of cell complexes and to highlight the conceptual differences using a concrete example. The revised text reads: "Cell complexes are higher-order network structures built from cells of different dimensions, such as nodes, edges, polygons,

polytopes, and higher-dimensional analogues [2–4]. Most studies on synchronization focus on the special case of simplicial complexes [5, 6], where each cell is a simplex. However, many real-world systems, such as social interactions or protein complexes, are better described by cell complexes, as their interactions are not naturally simplicial [2, 4, 7]. Such models can be formulated in arbitrary dimensions. However, many applications focus on the case where the dynamics is defined on edges and interactions occur via nodes and faces [8]."

- We have also revised the section "Properties of cell complexes" to provide a clearer and more structured introduction to the underlying concepts.

Fig. 2 is the only Figure that is completely clear and easy to understand. Nonetheless, it is not mentioned explicitly that the orientation is needed to have a base for the boundary operators, while, even to authors familiar with network science, this may look like a directionality.

Our reply: We thank the reviewer for this important clarification. We agree that the role of orientation may be easily misinterpreted as physical directionality, especially for readers familiar with network science. We have revised both the figure and the accompanying text to make this distinction explicit and improve clarity.

Changes to the manuscript:

- We have moved the respective figure to the beginning of the paper to support the introduction of the formalism.
- We have extended the figure to include the boundary matrices, making the correspondence between the geometric structure and its algebraic representation more transparent.
- We have added sample time series to motivate the analysis of phase-locked states.
- We have clarified the role of orientation in both the caption and the main text. The caption now states: "Arrows indicate the orientation used to define the boundary operators."
- In the main text, we added: "After fixing an orientation, their entries take values in $\{-1, 0, +1\}$, indicating whether a given $(n-1)$-cell appears in the boundary with positive or negative orientation, or not at all. The choice of orientation determines the signs in $\boldsymbol{B}_{[n]}$, but does not imply any physical directionality of the interactions."

It would greatly help if the structures of Fig. 2 would be used to explicitly compute the conditions for phase locking.

Our reply: We thank the reviewer for this very helpful suggestion. We agree that explicitly working out the phase-locking conditions for a concrete example greatly improves clarity, and we have revised the manuscript accordingly.

First, we have extended Fig. 2 (now Fig. 1) to include the boundary matrices and illustrative time series for selected phase-locked states, providing an intuitive entry point to the formalism.

Second, we have expanded the analysis of the hexagonal ring to serve as a coherent, step-by-step example demonstrating how the topological nonlinear Kirchhoff conditions are applied in practice.

Changes to the manuscript:

- The figure has been moved to the beginning of the manuscript and extended to include boundary matrices and representative time series.
- We now provide a detailed derivation of the phase-locking conditions for the hexagonal ring as a running example.
- In particular, we explicitly compute the relevant algebraic structures: the boundary operators are given in Fig. 1c; the kernel of $\boldsymbol{B}_{[1]}^{\top}$ is spanned by the harmonic vector $\mathbf{1}$, such that $\boldsymbol{C}_{[1]}^{\top} = \mathbf{1}$; and the kernel of $\boldsymbol{B}_{[2]}$ is trivial, implying $\boldsymbol{C}_{[2]} = \mathbf{0}$.

Not all readers may be familiar with the Topological Kuramoto. A brief recap and an explanation of what it means to project a cochain one dimension up or one dimension down should be given. Also, given that these are the variables that are then studied, some discussion of the dynamics of the latter could help contextualize the results.

Changes to the manuscript:

- We have split the first section of the Results into two parts: (i) "Synchronization on graphs and cell complexes", which provides an intuitive introduction for a general audience, and (ii) "Properties of cell complexes", which contains the mathematical details. Both sections have been substantially revised and extended.
- In the first section, we now explicitly introduce and explain the projection of variables to lower and higher dimensions, emphasizing its physical interpretation in terms of interactions across adjacent cells.
- We consistently use the term "interaction variables" for $\boldsymbol{\theta}^{[\pm]}$ and clarify their meaning directly after their definition: "The variable $\boldsymbol{\theta}^{[-]}$ measures phase differences across shared $(n-1)$-cell boundaries, while $\boldsymbol{\theta}^{[+]}$ measures phase accumulation around $(n+1)$-cells. For edge dynamics $(n = 1)$, these correspond to imbalances at nodes and circulations around faces, respectively."
- To further contextualize these variables, we have added examples of their dynamics $\boldsymbol{\theta}^{[\pm]}(t)$ in Fig. 3 and revised the corresponding discussion.

Some more details behind the rationale of the topological Kirchhoff condition could be given to guide the reader on what the authors are doing and why.

Our reply: We thank the reviewer for this important suggestion. We agree that the rationale behind the topological nonlinear Kirchhoff conditions should be made more transparent to guide the reader. We have therefore revised the corresponding section to better motivate the approach and to clearly explain both what is done and why.

In particular, we now begin with an intuitive explanation that connects Kirchhoff's voltage law (KVL) in linear circuits to its nonlinear generalization in the standard Kuramoto model, and then extend this reasoning to the topological Kuramoto model. This conceptual progression is illustrated in the revised Fig. 2, while the formal mathematical derivation is presented afterwards.

Changes to the manuscript:

- We now introduce the method through a clear two-step perspective: "To characterize all phase-locked states, we adopt a two-step approach. First, we determine candidate solutions by enforcing the local balance conditions in Eqs. (11)-(12). These conditions are linear in suitable auxiliary variables and define a low-dimensional solution space, with remaining degrees of freedom corresponding to circulations around cycles. In the second step, we impose nonlinear global consistency conditions that select those solutions that can be realized by actual phase variables."
- We then explain this construction using the example in Fig. 2, first recalling KVL and its nonlinear analogue in the standard Kuramoto model, before extending the argument to the topological setting.
- We have revised and extended Fig. 2 to support this step-by-step explanation and to make the underlying rationale more explicit (see also our replies above).

At least an explicit example of the matrices C should be given. Also, some more details on the kernels of the boundary operators and their relation with the kernel of the Hodge Laplacian and the Betti numbers could be given, so to have more intuition of what these quantities mean topologically.

Our reply: We thank the reviewer for this helpful suggestion. We agree that both an explicit example of the matrices $\boldsymbol{C}$ and a clearer discussion of their topological meaning improve the accessibility of the manuscript. We have therefore revised the text accordingly by including a concrete example and by expanding the discussion of the underlying algebraic and topological structures.

Changes to the manuscript:

- For the hexagonal ring example, we give the C matrices explicitly. We write "The kernel of the coboundary operator $\boldsymbol{B}_{[1]}^{\top}$ is spanned by the harmonic vector $\mathbf{1}$ such that $\boldsymbol{C}_{[1]}^{\top} = \mathbf{1}$. The kernel of the boundary operator $\boldsymbol{B}_{[2]}$ is trivial such that we can write $\boldsymbol{C}_{[2]} = \mathbf{0}$."

- We extended the respective paragraph as follows: "The relation $\boldsymbol{B}_{[n]}\boldsymbol{B}_{[n+1]} = 0$ leads to the Hodge decomposition [1], a discrete analogue of the Helmholtz decomposition from vector calculus. Any vector $\boldsymbol{x}_{[n]} \in \mathbb{R}^{N_{[n]}}$ can be uniquely decomposed as

$$\boldsymbol{x}_{[n]} = \boldsymbol{x}_{[n]}^{H} + \boldsymbol{B}_{[n]}^{\top}\boldsymbol{z}_{[n-1]} + \boldsymbol{B}_{[n+1]}\boldsymbol{z}_{[n+1]}. \tag{2}$$

The second term is a gradient-like component induced from lower dimensions, while the third is a curl-like component describing circulation around higher-dimensional cells. In addition, there is a harmonic component $\boldsymbol{x}_{[n]}^{H}$, which satisfies

$$\boldsymbol{B}_{[n]}\boldsymbol{x}_{[n]}^{H} = \boldsymbol{0}, \qquad \boldsymbol{B}_{[n+1]}^{\top}\boldsymbol{x}_{[n]}^{H} = \boldsymbol{0}.$$

Thus, it lies in the intersection of the kernels of $\boldsymbol{B}_{[n]}$ and $\boldsymbol{B}_{[n+1]}^{\top}$, and its dimension is given by the nth Betti number, i.e., the number of independent cycles (holes) in the complex. It corresponds to conserved, divergence-free flows that circulate along topological cycles without being generated by local gradients or curls, and persist due to the presence of holes or loops."

The division in partitions where the cosine is positive or negative is clever. But can the authors give more intuition of what it means? Again, an explicit example would help.

Our reply: We thank the reviewer for this insightful comment. We agree that the partitioning according to the sign of the cosine requires additional intuition and clarification. We have therefore revised the manuscript to better explain its meaning and implications, and to guide the reader through explicit examples.

Changes to the manuscript:

- We have added the following explanatory paragraph: "The partition is not uniquely determined by the dynamics, but must be chosen consistently with the reconstructed phases. Different partitions correspond to different branches of the inverse sine and therefore to distinct candidate solutions. Physically, the partition specifies how each phase difference is realized, i.e., whether it lies on the increasing or decreasing branch of the sine function, as determined by the sign of $\cos(\theta_i^{[\pm]})$."
- To clarify the implications, we now directly follow this discussion with: "Phase-locked states with $S^{\circ} = \emptyset$ are always linearly stable and are therefore especially relevant for applications. States with $S^{\circ} \neq \emptyset$ are typically, but not always, unstable. "
- We have added an explicit illustrative example: "For example, on a ring, choosing all cells in $S^{\bullet}$ corresponds to states in which all phase differences lie on the branch with $\cos(\theta_i) > 0$, i.e., $|\theta_i| < \pi/2$. In contrast, partitions containing elements of S° correspond to configurations where some phase differences lie on the branch with $\cos(\theta_i) \leq 0$. While such states are usually unstable, we will demonstrate an exception where stable phase-locked states with $S^{\circ} \neq \emptyset$ exist close to bifurcations."
- In the detailed ring example, we further emphasize this interpretation: "At this point, we recall the meaning of the partitions $S_{[0]}^{\bullet}$ and $S_{[0]}^{\circ}$: they label the two possible branches of the inverse sine appearing in the stationary equations. On cells in $S_{[0]}^{\bullet}$ we take $\theta_i^{[-]} = \arcsin(\psi_i^{[-]})$, whereas on cells in $S_{[0]}^{\circ}$ we take $\theta_i^{[-]} = \pi - \arcsin(\psi_i^{[-]})$. Thus, each partition represents a different candidate branch assignment for reconstructing a phase-locked state from a given solution $\boldsymbol{\psi}^{[-]}$. The physically relevant states will be obtained after solving the nonlinear Kirchhoff conditions."

The literature on the topic is not much and already a bit difficult. Using more standard terms, like n-simplices, n-chains, n-cells, n-cochains would help. Even with maybe a small dictionary at the beginning, so that every reader is on the same page. In fact, the notation is often inconsistent (e.g., sometimes "nodes", some others "vertices").

Our reply: We thank the reviewer for this helpful comment. We agree that clarity and consistency of terminology are particularly important in this area, where concepts from network science and algebraic topology intersect.

In response, we have carefully revised the manuscript to standardize notation and terminology throughout, while

maintaining accessibility for a broad audience. In particular, we adopt consistent terminology and clarify its relation to standard concepts from the literature.

While we appreciate the suggestion of including a dedicated dictionary, we instead introduce and clarify key terms at their first occurrence, in order to keep the presentation concise and integrated into the narrative.

Changes to the manuscript:

- We now use the term "node" consistently throughout the manuscript.
- We consistently use the term "boundary operator" instead of "boundary matrix".
- We consistently use the term "interaction variables" for $\boldsymbol{\theta}^{[\pm]}$ to emphasize their physical meaning, and clarify this directly after their definition: "The variable $\boldsymbol{\theta}^{[-]}$ measures phase differences across shared $(n-1)$-cell boundaries, while $\boldsymbol{\theta}^{[+]}$ measures phase accumulation around $(n+1)$-cells." (see also our reply above).
- In the discussion of Fig. 2, we now explicitly state that "we generalize the notion of winding numbers from graph cycles to higher-dimensional cycles of the cell complex."
- After Eq. (18), we have added the sentence "Mathematically, these variables parametrize contributions from the kernel of the boundary operators, corresponding to harmonic components in the Hodge decomposition."

Can the authors explain in details what are the conditions of Eq.(22)? This is what is then plot to show the main results, so it is important to have an intuition of what it means. Same goes for the later introduced variable ζ.

Our reply: We thank the reviewer for this important question. We agree that Eq. (22) and the variables $\boldsymbol{\zeta}^{[\pm]}$ are central to our results and require additional explanation and intuition. We have therefore revised the manuscript to provide both a clearer mathematical interpretation and a more explicit physical understanding of these quantities.

Changes to the manuscript:

- We have revised and extended the explanation of the variables $\boldsymbol{\zeta}^{[\pm]}$. The new version reads: "... where $\boldsymbol{\zeta}^{[\pm]}$ parametrize the space of homogeneous solutions. Mathematically, these variables parametrize contributions from the kernel of the boundary operators, corresponding to harmonic components in the Hodge decomposition. Physically, the parameters $\boldsymbol{\zeta}^{[\pm]}$ quantify the amplitude of circulating flows along independent cycles of the complex. They generalize loop currents in networks: different values of $\boldsymbol{\zeta}^{[\pm]}$ redistribute phase differences around cycles without violating local balance conditions."
- We have also revised and extended the discussion of Eq. (23) (Eq. (22) in the original manuscript) to clarify its meaning. In particular, we now state: "Equation (23) enforces global consistency of the reconstructed phase differences. The matrices $\boldsymbol{C}_{[n+1]}^{\top}$ and $\boldsymbol{C}_{[n]}^{\top}$ extract sums of phase differences along independent cycles in the corresponding higher- and lower-dimensional cycle spaces. The conditions therefore require that the total phase difference accumulated along each such cycle equals an integer multiple of 2π. The vectors $\boldsymbol{z}^{[\pm]}$ count how many times the phases wind around these cycles, with each component representing the winding number of one independent cycle. Importantly, $\boldsymbol{z}^{[\pm]} = \boldsymbol{0}$ indicates zero winding, but does not in general imply complete synchronization of the original phase variables $\boldsymbol{\theta}$.

  For a fixed partition, Eq. (23) becomes a nonlinear constraint on the parameters $\boldsymbol{\zeta}^{[\pm]}$ via the parametrization (18). It selects those candidate solutions for which there exists a phase vector $\boldsymbol{\theta}$ satisfying $\boldsymbol{\psi}^{[\pm]} = \sin(\boldsymbol{\theta}^{[\pm]})$. In this sense, Eqs. (23) generalize Kirchhoff's voltage law: the accumulated phase differences around each independent cycle must be equal to an integer multiple of 2π."

In Figs. 3 and 4 (as well as the others), it is very difficult to distinguish between filled and empty circles. In general, all Figures are extremely difficult to understand, and the captions do not help much in grasping what it is presented.

Our reply: We thank the reviewer for this important comment. We agree that the readability of the figures and the clarity of their captions are crucial, and we have revised both accordingly to improve accessibility and interpretation.

Changes to the manuscript:

- The filled and empty circle markers have been enlarged and their visual contrast improved to make them clearly distinguishable.
- In Fig. 3, we have added new panels **b,c,d**, showing time series corresponding to the stable phase-locked states identified in panel **a**, thereby providing a more direct dynamical interpretation.
- We have thoroughly revised the captions of Figs. 3 and 4, as well as most others, to explicitly describe the content of each panel, including axes, curves, and markers, and to better explain the distinction between stable and unstable solution branches.

The lower panel in Fig. 4 is not clear.

Our reply: We thank the reviewer for pointing this out. We agree that the lower panel was not sufficiently clear, and we have revised Fig. 4b to improve its readability and interpretation.

Changes to the manuscript:

- In Fig. 4b, we have refined the legend to more clearly distinguish the different solution branches and added an explicit annotation indicating the saddle-node bifurcation.
- We have increased the size of the panel and adjusted the layout to improve readability.
- We have revised the caption to clarify the role of ω_0 as the bifurcation control parameter.

It is interesting how heterogeneous frequencies can be used to control the emergence of multistability. Could the authors make an explicit example?

Our reply: We thank the reviewer for this insightful suggestion. We agree that the role of heterogeneous natural frequencies as a control mechanism for multistability should be illustrated more explicitly. While such examples were already present in the manuscript (for the ring and the cube), we have revised the text to highlight and explain them more clearly.

Changes to the manuscript:

- In the hexagonal ring example, we now explicitly present how heterogeneous frequencies control the emergence and stability of phase-locked states. The revised paragraph reads: "Next, we consider heterogeneous natural frequencies, taking

  $$\boldsymbol{\omega} = (0, 0, 0, 0, +\omega_0, -\omega_0). \tag{3}$$

  This choice breaks the underlying permutation symmetry of the ring by singling out one specific cell and we will therefore refer to it as asymmetric. The scalar parameter ω_0 acts as a control parameter for the bifurcation structure shown in Fig. 4. We limit our exploration to two partitions, $S^{\circ}_{[0]} = \emptyset$ and $S^{\circ}_{[0]} = [5, 6]$.

  In Fig. 4**a** we plot the Kirchhoff condition (26), normalized by 2π, as a function of $\zeta^{[-]}$ for chosen values of ω_0. For small ω_0, the partition $S^{\circ}_{[0]} = \emptyset$ supports stable phase-locked solutions, whereas the partition $S^{\circ}_{[0]} = [5, 6]$ is typically associated with unstable ones.

  As ω_0 is increased, however, the frequency heterogeneity qualitatively changes the solution structure. In a narrow interval of ω_0, the branch associated with $S^{\circ}_{[0]} = \emptyset$ disappears, while the partition $S^{\circ}_{[0]} = [5, 6]$ supports two distinct solutions, one stable and one unstable. Thus, heterogeneous intrinsic frequencies act as a control mechanism that selects which partition carries the stable phase-locked state: stability has shifted from the all-normal partition to a mixed partition, $S^{\circ}_{[0]} = [5, 6]$. Increasing ω_0 further, the stable and unstable $S^{\circ}_{[0]} = [5, 6]$ branches collide and annihilate in a saddle-node bifurcation, as shown in Fig. 4**b**."
- In the section on Platonic solids, we clarify the corresponding mechanism in a higher-dimensional example: "We finally demonstrate how heterogeneity controls these states using the cube. For $\boldsymbol{\omega} = \mathbf{0}$, nine stable states coexist. Introducing heterogeneous frequencies breaks symmetry and progressively eliminates solutions, with states disappearing in symmetric pairs or triplets (Fig. **??**). Thus, intrinsic heterogeneity controls the number of stable phase-locked states."

The authors claim that cell complexes support richer multistability than simplicial complexes of the same dimension. First of all, we are not entirely sure about this, as from Fig. 6 it seems that the cube (cell complex) has the same number of stable state than the octahedron (simplicial complex), and the dodecahedron (cell complex) the same as the icosahedron (simplicial complex). If we are wrong and did not understand correctly, can the authors explain the Figure? Assuming that their claim is correct: (1) is it valid for any n-cochain? (2) how is it compatible with the fact that, for 1-cochains, synchronization is obstructed on simplicial complexes while not on cell complexes? (so, one would assume a richer dynamics where there cannot be synchronization).

Our reply: We thank the reviewer for this careful and insightful comment. We agree that the comparison between cell and simplicial complexes is more subtle than suggested by the original formulation. In particular, as noted by the reviewer, Fig. 6 does not show a straightforward difference in the number of stable states between corresponding examples such as the cube and the octahedron or the dodecahedron and the icosahedron.

Our intention was to highlight a structural effect: the emergence of multistability is governed by the boundary size, and the condition $m_k \geq 5$ is typically realized with fewer cells in cell complexes than in simplicial complexes. For instance, comparable multistability can arise in a dodecahedron (12 pentagonal faces) and an icosahedron (20 triangular faces), reflecting that larger boundary sizes must be built up differently in the two settings.

We agree, however, that this correspondence is indirect and that the original wording may suggest a more immediate comparison than is warranted. We have therefore revised the manuscript to avoid the potentially misleading comparative phrasing and to better emphasize the underlying structural mechanism.

Changes to the manuscript:

- We have reformulated the corresponding statement in the introduction to avoid a direct comparison in terms of "richer" multistability. It now reads: "Using rings, Platonic solids, and regular simplices as illustrative examples, we uncover a universal rule: boundaries must have at least five elements for multistability to arise."

The authors seem to use "simplex" and "simplicial complex" to mean the same thing, but this causes confusion. A simplex is just one part of the structure, e.g., a 0-simplex is a node, a 1-simplex is a link, etc., while the structure is a simplicial complex. This should be fixed. With such definitions, Fig. 8 results extremely confusing.

Our reply: We thank the reviewer for pointing this out. We agree that our original wording did not clearly distinguish between a simplex and a simplicial complex, which may cause confusion. We have revised the manuscript to consistently use the correct terminology and to clarify the structures under consideration.

Changes to the manuscript:

- We have revised the opening paragraph of the subsection to explicitly distinguish between a simplex and the simplicial complex it generates. It now reads: "We now consider simplicial complexes generated by a single d-simplex, i.e., a d-simplex together with all of its boundary simplices of dimensions $n = d-1, d-2, \ldots, 0$. For example, a tetrahedron (3-cell) gives rise to a simplicial complex consisting of its faces (2-cells), edges (1-cells), and nodes (0-cells). We then fix a dimension $n \leq d-1$ and analyze phase-locked states of the topological Kuramoto model defined on n-simplices interacting via simplices of dimension $n \pm 1$."
- We have revised the caption of the corresponding table (former Fig. 8) to reflect this terminology: "**Stable phase-locked states for simplicial complexes generated by a single d-simplex, for $d \leq 5$.** The first column lists each simplicial complex together with its cell counts. The table gives, for each admissible cochain dimension n, the number of stable all-normal states and the associated winding numbers. Here $\mathbf{0}_m$ denotes the zero vector of dimension m. Entries with more than one stable state are shown in bold."

Minor comments:

line 320: does not corresponds;

i.e. should be followed by a comma.

Our reply: We thank the reviewer for pointing out these typographical errors.

Changes to the manuscript:

- We have corrected all instances of these errors throughout the manuscript.

REVIEWER #2

I co-reviewed this manuscript with one of the reviewers who provided the listed reports. This is part of the Nature Communications initiative to facilitate training in peer review and to provide appropriate recognition for Early Career Researchers who co-review manuscripts.

Our reply: We thank the co-reviewer for their careful reading and helpful comments.

Changes to the manuscript: See above.

REVIEWER #3

The authors consider a topological Kuramoto model generalized to cell complexes. They present a method for identifying all phase-locked states and their stability. They systematically applied their method to structures of increasing dimensions, and show that multistability requires a minimum number of boundary cells in the structure, and that multistability can be reduced by introducing asymmetry in the natural frequencies.

I found the results very interesting, novel, and strong. The text is well written, although a bit on the technical side, and I appreciate the effort in making the figures. I believe the method to identify the phase-locked states, and the results on multistability, will have an impact on future research in the area. Below, I mainly detail what was less clear to me, with suggestions to further improve the clarity and interpretation of some of the key mechanisms/variables, which whill hopefully help the manuscript have the impact I expect it will.

Our reply: We thank the reviewer for the very positive assessment of our work and for recognizing the novelty and potential impact of our results. We also appreciate the constructive suggestions aimed at improving clarity and accessibility.

Changes to the manuscript:

- In response, we have revised the manuscript to improve readability and interpretation of the key concepts. In particular, we have restructured the presentation of the Results section, added a new introductory section with a more intuitive discussion of the model and phase-locked states, clarified the role of key variables and mechanisms throughout, and improved the figures and their captions to better guide the reader.

A. Major comments

The text is technical, which is not a problem, because it shows strong analytical results. However some key aspects could be clarified:

l162: define what phase-locked states are in the context of the topological kuramoto (and how they differ from the standard Kuramoto). They are central to the paper but not defined.

Our reply: We thank the reviewer for this important comment. We agree that phase-locked states are central to the paper and should be introduced more clearly. In the revised manuscript, we now adopt a two-step approach, first providing an intuitive introduction based on examples, and then giving a precise mathematical definition.

Changes to the manuscript:

- In the section "Synchronization on graphs and cell complexes" we now introduce phase-locked states in an intuitive way using explicit examples: In Figure 1, "**e,f** Exemplary time series of the phase variables $\theta_{[}j,k](t)$ associated to the edges $[j,k]$ for both the ring and the cube. The system converges to different phase-locked states depending on the respective initial conditions."
- In the subsequent section, we then provide a precise mathematical definition and discussion: " Our analysis is based on the interaction variables $\boldsymbol{\theta}^{[-]}$ and $\boldsymbol{\theta}^{[+]} = \boldsymbol{B}_{[n+1]}^{\top}\boldsymbol{\theta}$, whose dynamics is given in Eq. (8). Setting the time derivatives to zero yields the nonlinear algebraic conditions [...]. A subtle point is that the conditions $\frac{d}{dt}\boldsymbol{\theta}^{[\pm]} = \mathbf{0}$ do not fully fix the original variables $\boldsymbol{\theta}$. In particular, $\boldsymbol{\theta}$ may still contain a harmonic component that does not contribute to $\boldsymbol{\theta}^{[-]}$ or $\boldsymbol{\theta}^{[+]}$. This component affects the dynamics only in a trivial way, corresponding to a global or circulating shift of the phases that does not enter the interaction terms. As a result, it is irrelevant for synchronization, and we refer to solutions of Eqs. (11)-(12) as phase-locked states rather than strict fixed points."

ζ is the key control parameter in the main figures (figs 3, 4, 6, 7), but is only introduced as "vectors of free coefficients which parametrize the homogeneous solution space". Add a "physical" interpretation of their origin and of what they control.

Our reply: We thank the reviewer for this important comment. We agree that the role of the parameters $\boldsymbol{\zeta}^{[\pm]}$ should be better motivated and physically interpreted, in particular given their central role in the figures.

Changes to the manuscript:

- We have revised the introduction of the variables $\boldsymbol{\zeta}^{[\pm]}$ to include both a mathematical and a physical interpretation. In particular, we now write: "...where $\boldsymbol{\zeta}^{[\pm]}$ parametrize the space of homogeneous solutions. Mathematically, these variables parametrize contributions from the kernel of the boundary operators, corresponding to harmonic components in the Hodge decomposition. Physically, the parameters $\boldsymbol{\zeta}^{[\pm]}$ quantify the amplitude of circulating flows along independent cycles of the complex. They generalize loop currents in networks: different values of $\boldsymbol{\zeta}^{[\pm]}$ redistribute phase differences around cycles without violating local balance conditions."
- We also clarify their role as control parameters in the solution space by emphasizing that, for a fixed partition, the nonlinear Kirchhoff conditions impose constraints on $\boldsymbol{\zeta}^{[\pm]}$, thereby selecting the admissible phase-locked states. This interpretation is now made explicit in the discussion of the main equations and in the description of the figures.

l 341 and 358 and throught: w=0 and w!=0 are described as symmetric and asymmetric. Please explain why. w is used as a control parameter in key results (eg figs 4, 7) and it should be clear.

Our reply: We thank the reviewer for this important comment. We agree that the terminology "symmetric" and "asymmetric" should be clearly justified, especially since the frequency vector $\boldsymbol{\omega}$ plays a central role as a control parameter in our analysis.

Changes to the manuscript:

- We now explicitly explain the terminology when first introducing the parameter $\boldsymbol{\omega}$. In particular, we write: "We start by exploring the fully symmetric case when $\boldsymbol{\omega} = 0$ and $\boldsymbol{K}_{[1]} = \mathbb{1}$. We refer to this case as symmetric because it preserves the full permutation symmetry of the underlying complex. "
- Later, we consider heterogeneous natural frequencies and add: "Next, we consider heterogeneous natural frequencies, taking

$$\boldsymbol{\omega} = (0, 0, 0, 0, +\omega_0, -\omega_0). \tag{4}$$

This choice breaks the underlying permutation symmetry of the ring by singling out one specific cell and we will therefore refer to it as asymmetric."

- We have made this interpretation consistent throughout the manuscript and clarified the role of $\boldsymbol{\omega}$ as a control parameter. In particular, we now state: "The scalar parameter ω_0 acts as a control parameter for the bifurcation structure shown in Fig. 4."
- We further emphasize that changing $\boldsymbol{\omega}$ shifts the balance conditions and thereby alters the set of admissible phase-locked states. This is illustrated explicitly in the ring and cube examples, where specific choices of $\boldsymbol{\omega}$ break the geometric symmetry by singling out selected cells.

Key results and figures discuss different possible partitions. It was not fully clear to me what those different partitions represent ("physically"). I initially thought was unique and defined based on the sign of cos(theta).

Our reply: We thank the reviewer for this important comment. We agree that the meaning of the partitions and their physical interpretation were not sufficiently clear in the original version.

Changes to the manuscript:

- We now explicitly clarify that the partitions are not uniquely determined a priori, but correspond to different choices of branches when inverting the sine function. In particular, we write: "The partition is not uniquely determined by the dynamics, but must be chosen consistently with the reconstructed phases. Different partitions correspond to different branches of the inverse sine and therefore to distinct candidate solutions."
- We have added a physical interpretation to guide the reader: "Physically, the partition specifies how each phase difference is realized, i.e., whether it lies on the increasing or decreasing branch of the sine function, as determined by the sign of $\cos(\theta_i^{[\pm]})$."
- To address the reviewer's point directly, we now emphasize that the partition is not fixed beforehand (e.g., by $\cos(\theta)$), but is part of the solution construction. Only after solving the nonlinear consistency conditions can one determine which partitions correspond to valid phase-locked states.
- We have also added illustrative explanations in the ring example, where different partitions are explicitly linked to different candidate states and their stability properties.

z is introduced as " a vector of integers" and "in the case of ordinary graphs, as winding vectors" (l 263). This interpretation is never used in the description of the results also z is often explicitly given in many cases of "Class 3: simplexes". At some point I even found myself wondering if z = (0, 0, 0) was corresponding to full sync (which it does not). So remind the readers and help them interpret those values.

In the discussion, consider also adding some interpretations to the results based on the above.

Our reply: We thank the reviewer for this important comment. We agree that the interpretation of the winding vectors $\boldsymbol{z}^{[\pm]}$ was not sufficiently emphasized in the original version, and that this may lead to confusion when interpreting the results.

Changes to the manuscript:

- We have expanded the discussion when introducing the winding numbers to clarify their meaning and role. In particular, we now write: "Equation (23) enforces global consistency of the reconstructed phase differences. The matrices $\boldsymbol{C}_{[n+1]}^{\top}$ and $\boldsymbol{C}_{[n]}^{\top}$ extract sums of phase differences along independent cycles in the corresponding higher- and lower-dimensional cycle spaces. The conditions therefore require that the total phase difference accumulated along each such cycle equals an integer multiple of 2π. The vectors $\boldsymbol{z}^{[\pm]}$ count how many times the phases wind around these cycles, with each component representing the winding number of one independent cycle. Importantly, $\boldsymbol{z}^{[\pm]} = \boldsymbol{0}$ indicates zero winding, but does not in general imply complete synchronization of the original phase variables $\boldsymbol{\theta}$."
- To address the reviewer's point directly, we now explicitly clarify the interpretation of $\boldsymbol{z}^{[\pm]}$ in the examples. In the discussion of simplicial complexes, we write: "The winding vectors characterize topological winding in the interaction variables $\boldsymbol{\theta}^{[\pm]}$."

- We have also revised the discussion of the results to more consistently remind the reader of this interpretation when presenting specific values of $\boldsymbol{z}^{[\pm]}$, in order to facilitate the physical interpretation of the different solution branches.

l 549: " we showed that multistability emerges when boundaries contain more than four cells". This is very interesting. Do you have any idea of where this number 4 comes from? Why 4?

Our reply: We thank the reviewer for highlighting this point. We agree that the origin of this threshold deserves a clearer explanation.

In fact, a nonzero winding requires the phase differences accumulated around a cycle to sum to at least 2π, but each contribution is less than $\frac{\pi}{2}$, so at least five boundary cells are needed for robust multistability. We explain this fact in detail in the revised version.

Changes to the manuscript:

- We have expanded the discussion and now present it in a dedicated subsection, "Universal boundary-size rule for multistability". In particular, we added the following explanation: "Geometrically, Eq. (24) expresses a phase-budget constraint. Each reconstructed phase difference contributing to the cycle sum is bounded in magnitude by $\pi/2$, while a nonzero winding requires an accumulated phase difference of at least 2π. Consequently, at least five contributions are needed to realize nontrivial winding. Boundaries with fewer than five cells cannot support multistability, whereas larger boundaries can."

Where relevant, compare your results with the literature on hypergraphs.

Our reply: We thank the reviewer for this important suggestion. We agree that hypergraphs provide an alternative and widely studied framework for modeling higher-order interactions, and we have clarified their relation to our work in the revised manuscript.

While both approaches aim to describe higher-order interactions, they capture different structural aspects. Cell complexes provide additional geometric and topological structure through boundary operators and orientation, which enable the definition of flows, conservation laws, and topological constraints such as winding numbers. These concepts are central to our analysis. In contrast, hypergraphs encode higher-order interactions as sets of nodes, but do not specify how these interactions are organized across dimensions or how they share boundaries.

As a result, a direct comparison of results is not straightforward, since the underlying mathematical structures and notions of dynamics differ. We have clarified this distinction in the introduction.

Changes to the manuscript:

- We have added the following sentence to the introduction: "Alternative representations of higher-order interactions, such as hypergraphs, have also been explored, but they capture different structural aspects of complex systems [9, 10]."

B. Minor comments

l49: "revealing universal features absent in conventional networks, such as abrupt synchronization". This sounds mistaken, abrupt synchronization has been shown also in non topological networks.

Our reply: We thank the reviewer for pointing out this imprecise wording. We agree that abrupt synchronization transitions are not exclusive to topological or higher-order systems.

Changes to the manuscript:

- We have rephrased the sentence from "has become a paradigmatic framework, revealing universal features absent in conventional networks, such as abrupt synchronization transitions [11, 12]." to "revealing novel synchronization phenomena, including abrupt transitions in higher-order settings [11, 12]."

l90-112: Cell complexes are not common as simplicial complexes or hypergraphs in synchronization literature and need to be introduced with more precision or care. There is not explicit definition of what a cell complex is. Maybe also add a comparison with simplcial complexes and hypergraphs too where possible.

l97: "but many real-world systems are better described by cell complexes" add a reference and examples, and clarify why they are better described by cell complexes

Our reply: We thank the reviewer for these two helpful comments, which we address at the same time. We agree that cell complexes require a more careful introduction, especially given that they are less commonly used in the synchronization literature. In the revised manuscript, we have expanded the introduction of cell complexes and clarified their relation to simplicial complexes. We also briefly mention alternative representations such as hypergraphs without going into technical detail.

Changes to the manuscript:

- In the introduction, we now clarify the relation to simplicial complexes and explain why cell complexes are sometimes more appropriate: " Cell complexes are higher-order network structures built from cells of different dimensions, such as nodes, edges, polygons, polytopes, and higher-dimensional analogues [2–4]. Most studies on synchronization focus on the special case of simplicial complexes [5, 6], where each cell is a simplex. However, many real-world systems, such as social interactions or protein complexes, are better described by cell complexes, as their interactions are not naturally simplicial [2, 4, 7]."
- In the section "Properties of cell complexes" we have added a more explicit explanation: "Cell complexes are geometrical objects built from elements of different dimension n. The building blocks are called n-cells and include nodes (0-cells), edges (1-cells), faces (2-cells), volumes (3-cells), and higher-dimensional analogues. Two examples are shown in Fig. 1**a,b**: a hexagonal face ($n = 2$) with six edges and six nodes, and a cube ($n = 3$) with six faces, twelve edges, and eight nodes.

  An important subclass is given by *simplicial complexes*, where every n-cell is an n-simplex, i.e., a cell spanned by $n + 1$ nodes (e.g., triangles for $n = 2$ and tetrahedra for $n = 3$). However, many systems involve interactions over loops or surfaces that are not fully connected. Representing such structures as simplicial complexes requires artificial triangulations, introducing additional cells and incidence relations (e.g., a square face must be decomposed into two triangles). We therefore work in the more general setting of cell complexes."
- We also added a brief remark in the introduction noting that alternative representations such as hypergraphs capture different structural aspects of higher-order interactions.

l229: is there a reason/name why ψ_{sp} is called "sp"?

Our reply: We thank the reviewer for pointing this out. The subscript "sp" was intended to denote a particular solution, but this notation was unclear.

Changes to the manuscript:

- We have corrected this throughout the manuscript by renaming the variable to ψ_{p}, following standard notation for particular solutions.

l249: I only understood later (first example) that the notation full/empty circle based on the cos(theta) sign was because that sign determines stable/unstable (Eq 11). Maybe remind it there.

Our reply: We thank the reviewer for this helpful suggestion. We agree that the connection between the sign of $\cos(\theta)$, the partition, and stability should be made explicit earlier.

Changes to the manuscript:

- Right after introducing partitions, we now write: “The partition is not uniquely determined by the dynamics, but must be chosen consistently with the reconstructed phases. Different partitions correspond to different branches of the inverse sine and therefore to distinct candidate solutions. Physically, the partition specifies how each phase difference is realized, i.e., whether it lies on the increasing or decreasing branch of the sine function, as determined by the sign of $\cos(\theta_i^{[\pm]})$.

  Phase-locked states with $S^\circ = \emptyset$ are always linearly stable and are therefore especially relevant for applications. States with $S^\circ \neq \emptyset$ are typically, but not always, unstable. For example, on a ring, choosing all cells in $S^\bullet$ corresponds to states in which all phase differences lie on the branch with $\cos(\theta_i) > 0$, i.e., $|\theta_i| < \pi/2$. In contrast, partitions containing elements of S° correspond to configurations where some phase differences lie on the branch with $\cos(\theta_i) \leq 0$. While such states are usually unstable, we will demonstrate an exception where stable phase-locked states with $S^\circ \neq \emptyset$ exist close to bifurcations (cf. Fig. 4). ”

l258-260: ”The question whether a solution candidate (16) exists (...) can thus be formulated as [Eq 21].” is potentially confusing. If Eq 21 is satisfied, I’m guessing that it means that a ”a candidate solution (16) can be expressed as (12)”, and not the opposite (the phrasing does not specify which holds). If so, rephrase accordingly to make that explicit.

Our reply: We thank the reviewer for pointing out this ambiguity. We agree that the original phrasing did not clearly specify the logical relation between the old Eq. (21) and the realizability of candidate solutions.

We have revised the manuscript to make this point explicit. In particular, we now explicitly write: “A candidate solution (18) corresponds to a valid phase-locked state if and only if it can be expressed in the form (14).” This is then directly linked to Eq. (23), which encodes this condition in terms of orthogonality to the kernel of the boundary operators.

Furthermore, we have restructured the presentation of the topological nonlinear Kirchhoff conditions to improve clarity. We now first introduce the two-step conceptual framework, distinguishing between linear balance conditions and nonlinear global consistency constraints, and then provide the precise mathematical formulation. We believe that this revision removes the ambiguity and makes the logical structure of the argument explicit.

Changes to the manuscript: We have revised the section introducing the topological nonlinear Kirchhoff conditions as follows:

- In the introduction we now write “To characterize all phase-locked states, we adopt a two-step approach. [...] In the second step, we impose nonlinear global consistency conditions that select those solutions that can be realized by actual phase variables.”
- We then describe the global consistency conditions using the new Fig. 2, first for the standard Kuramoto model and then for the topological Kuramoto model.
- In the mathematical part we provide the precise formulation: “A candidate solution (18) corresponds to a valid phase-locked state if and only if it can be expressed in the form (14). Equivalently, the reconstructed $\boldsymbol{\theta}^{[\pm]}$ must lie in the image of the appropriate boundary or coboundary operators. Since a vector lies in the image of a matrix if and only if it is orthogonal to the kernel of the transpose matrix, this condition can be written as ...”

l268-281: ”To summarize, we propose the following algorithm” it’s not immediately clear that 4 points below are a summary of what was described before. ”2. Choose a partition” but I thought the partition was unique and defined based on the sign of cos(theta)? Point 4: When is it not necessary to the check the stability from the Jacobian?

Our reply: We thank the reviewer for these helpful comments. We agree that the original formulation of the algorithm and the discussion of partitions and stability could lead to confusion and we have revised the manuscript accordingly.

First, we clarify that the algorithm is not a summary but a direct procedural consequence of the framework developed above.

Second, regarding the partition, we emphasize that it is not uniquely determined a priori. Instead, different partitions correspond to different branches of the inverse sine and must be considered separately when constructing candidate solutions. The physically realized partition is then determined a posteriori by consistency with the reconstructed phases.

Third, we agree that the remark about stability in the original version was potentially misleading. In the revised manuscript, we have simplified the presentation and now consistently require stability to be checked via the Jacobian.

Changes to the manuscript:

- Right after introducing partitions, we now write: "The partition is not uniquely determined by the dynamics, but must be chosen consistently with the reconstructed phases. Different partitions correspond to different branches of the inverse sine and therefore to distinct candidate solutions. Physically, the partition specifies how each phase difference is realized, i.e., whether it lies on the increasing or decreasing branch of the sine function, as determined by the sign of $\cos(\theta_i^{[\pm]})$."
- We now introduce the algorithm with the sentence "This framework yields the following procedure for computing all phase-locked states."
- We have rephrased the algorithm steps for clarity and brevity as follows:
  1. Compute candidate solutions using Eq. (18), subject to $|\boldsymbol{\psi}^{[\pm]}| \leq 1$.
  2. Choose a partition $S^{\bullet}, S^{\circ}$ specifying the branch of the inverse sine for each component.
  3. Solve Eq. (23) for integer winding vectors $\boldsymbol{z}^{[\pm]}$ consistent with the chosen partition.
  4. Check linear stability via the Jacobian $\boldsymbol{J}$.

l337: "the of the" missing word, probably kernel?

Our reply: We thank the reviewer for catching this typographical error.

Changes to the manuscript:

- We have corrected the sentence by adding the missing word "kernel".

l330: "solutions candidates" → "solution candidates" or maybe even better "candidate solutions" throughout

Our reply: We thank the reviewer for this helpful suggestion and agree with the improved wording.

Changes to the manuscript:

- We now use the term "candidate solutions" consistently throughout the manuscript.

Fig 3: The caption should state what the x- and y- variables are (and what they represent), even if state in the main text.

Our reply: We thank the reviewer for this helpful suggestion and we have revised the caption accordingly.

Changes to the manuscript:

- We have revised the caption of Fig. 3 to explicitly state the quantities shown on the axes and their interpretation. It now reads: "**a** The normalized Kirchhoff condition $C_{[1]}^{\top} f_{[0]}(\psi^{[-]})/(2\pi)$ as a function of the scalar generalized loop flow amplitude parameter $\zeta^{[-]}$, shown for the distinct multiset partitions of $S_{[0]} = S_{[0]}^{\bullet} \cup S_{[0]}^{\circ}$. Intersections with integer values on the vertical axis correspond to phase-locked solutions with winding number $z^{[-]}$. Filled markers denote linearly stable solutions and open markers denote unstable solutions. All partitions except $|S_{[0]}^{\bullet}| = |S_{[0]}^{\circ}| = 3$ admit phase-locked states. In the all-normal case $|S_{[0]}^{\bullet}| = 6$, $|S_{[0]}^{\circ}| = 0$, three stable solutions coexist, corresponding to winding numbers $z^{[-]} = -1, 0, 1$. **b, c, d** Time series $\theta_i^{[-]}(t)$ for the six components of the lower interaction variables, obtained from perturbed initial conditions near the three stable all-normal branches. Dashed horizontal lines indicate the corresponding stationary values $\theta_i^{[-]} = \arcsin(\zeta^{[-]}) = 2\pi z^{[-]}/6$. The trajectories converge to the three coexisting states with winding numbers **b** $z^{[-]} = -1$, **c** $z^{[-]} = 0$, and **d** $z^{[-]} = 1$."

Fig 8: Would be most naturally displayed as a table (in text, rather than as an figure/image)

Our reply: We thank the reviewer for this helpful suggestion and agree that a table provides a clearer presentation.

Changes to the manuscript:

- We have converted this figure, together with the list from the main text, into a table (now Table 1) in the revised manuscript.

me being picky: text sub/superscripts should generally not be in italic/math mode. Use down *instead of down for example.*

Our reply: We thank the reviewer for this helpful remark and agree that text in subscripts and superscripts should not be typeset in math italics.

Changes to the manuscript:

- We have revised the manuscript accordingly and now use upright text in such cases (e.g., via `\rm` or `\text`), avoiding math italics in subscripts and superscripts.

REVIEWER #4

Potentially Important steps on extending the analysis of topological Kuramoto models on simplicial complexes to cell complexes.

Our reply: We thank the reviewer for this positive assessment of the scope and relevance of our work.

I think the image in Figure 1 is confusing and possibly inaccuarate whe interpreted in a natural way. If the symbols ψ_i are called phase differences (as the caption says), then it should be their algebraic sum that equals $2z\pi$ – not their arcsin.

Our reply: We thank the reviewer for pointing out this important issue. We agree that the original figure and caption could lead to a misleading interpretation. We have completely revised the figure and the caption, and we provide additional explanations in the main text.

In fact, the variables are defined as $\psi_i^{[\pm]} = \sin(\theta_i^{[\pm]})$ such that one needs the arcsin to recover the phase differences. The key advantage is that the stationarity conditions become linear in the variables $\psi_i^{[\pm]}$, at the expense of introducing nonlinear cycle constraints.

Changes to the manuscript:

- We have completely redesigned the conceptual figure (now Fig. 2) and rewritten the caption to clarify the meaning of all variables and the logical steps of the construction.
- We have restructured the presentation by first introducing Kirchhoff's voltage law (KVL), then its nonlinear generalization in the standard Kuramoto model, and finally its extension to the topological Kuramoto model. This step-by-step approach avoids ambiguities in interpretation.
- In the revised figure, the interaction variables $\psi_i^{[\pm]}$ are shown explicitly for a simple example (the tetrahedron), making their role and interpretation transparent.
- We have rewritten the corresponding section of the text to consistently distinguish between phase differences and their sine values, and to explain how the nonlinear Kirchhoff conditions arise from reconstructing phases via the inverse sine.

- We describe explicitly how the step from $\theta_i^{[\pm]}$ to $\psi_i^{[\pm]}$ changes the problem: "The stationarity conditions then become linear in ψ_i, while Kirchhoff's voltage law is replaced by a nonlinear condition involving the inverse sine."

I am also confused about the use of the arcsin function. I assume the document means the principal branch in $[-pi/2, pi/2]$, but are counterclockwise angular difference always guaranteed to take value in that interval?

Our reply: We thank the reviewer for raising this important point. We agree that the role of the arcsin function and its branches required clarification and we have revised the manuscript to make this explicit.

In fact, both branches of the arcsin are taken into account in our approach as both lead to different branches of the solution. However, the principal branch is most important for applications, as it leads to linearly stable phase-locked states under the normal stability condition. We have extended the discussion in the manuscript.

Moreover, one has to distinguish between the two branches for every cell separately. This is systematically handled by decomposing the set of edges into two subsets $S^\bullet$ and S°. We discuss this decomposition in more detail in the revised manuscript.

Changes to the manuscript:

- We have revised and extended the description of the second step of our method where we introduce the handling of the two branches of the arcsin: "In the second step, we generalize Kirchhoff's voltage law and select valid phase-locked states. In other words, we determine whether a candidate solution (18) can be represented in the form (14). This requires reconstructing $\boldsymbol{\theta}^{[\pm]}$ from $\boldsymbol{\psi}^{[\pm]}$. If Eq. (14) admits a solution, each component has two possible branches, given by $\arcsin(\cdot)$ and $\pi - \arcsin(\cdot)$. To keep track of these two possibilities, we partition the sets of cells $S_{[n+1]}$ and $S_{[n-1]}$ according to the sign of $\cos\left(\theta_i^{[\pm]}\right)$. If the cosine is positive, we define the inverse

$$f^\bullet(\psi_i^{[\pm]}) = \arcsin(\psi_i^{[\pm]}) \tag{5}$$

up to integer multiples of 2π and collect the respective cells in the sets $S^\bullet_{[n+1]}$ and $S^\bullet_{[n-1]}$. Likewise, if the cosine is not positive, we define the inverse

$$f^\circ(\psi_i^{[\pm]}) = \pi - \arcsin(\psi_i^{[\pm]}) \tag{6}$$

up to integer multiples of 2π and collect the respective cells in the sets $S^\circ_{[n+1]}$ and $S^\circ_{[n-1]}$."

- Furthermore, we have added the following explanatory paragraph: "The partition is not uniquely determined by the dynamics, but must be chosen consistently with the reconstructed phases. Different partitions correspond to different branches of the inverse sine and therefore to distinct candidate solutions. Physically, the partition specifies how each phase difference is realized, namely whether it lies on the increasing or decreasing branch of the sine function, as determined by the sign of $\cos(\theta_i^{[\pm]})$."

- To clarify the implications, we now directly follow this discussion with: "Phase-locked states with $S^\circ = \emptyset$ are always linearly stable and are therefore especially relevant for applications. States with $S^\circ \neq \emptyset$ are typically, but not always, unstable."

- We have added an explicit illustrative example: "For example, on a ring, choosing all cells in $S^\bullet$ corresponds to states in which all phase differences lie on the branch with $\cos(\theta_i) > 0$, i.e., $|\theta_i| < \pi/2$. In contrast, partitions containing elements of S° correspond to configurations where some phase differences lie on the branch with $\cos(\theta_i) \leq 0$. While such states are usually unstable, we will demonstrate an exception where stable phase-locked states with $S^\circ \neq \emptyset$ exist close to bifurcations."

- In the detailed ring example, we further emphasize this interpretation: "At this point, we recall the meaning of the partitions $S^\bullet_{[0]}$ and $S^\circ_{[0]}$: they label the two possible branches of the inverse sine appearing in the stationary equations. On cells in $S^\bullet_{[0]}$ we take $\theta_i^{[-]} = \arcsin(\psi_i^{[-]})$, whereas on cells in $S^\circ_{[0]}$ we take $\theta_i^{[-]} = \pi - \arcsin(\psi_i^{[-]})$. Thus, each partition represents a different candidate branch assignment for reconstructing a phase-locked state from a given solution $\boldsymbol{\psi}^{[-]}$. The physically relevant states will be obtained after solving the nonlinear Kirchhoff conditions."

The presentation/results on phase locking is very technical and hard to follow. There are many variables (typically without an intuitive name), many intermediate steps, no example cell complex and corresponding Kuramoto, and overall limited insight into the big picture.

Our reply: We thank the reviewer for this important comment. We agree that the original presentation was too technical and did not sufficiently guide the reader through the main ideas and mechanisms. Improving clarity and accessibility has been a central focus of our revision.

Changes to the manuscript: We have substantially revised the manuscript to provide a clearer and more intuitive presentation of the results:

- We have restructured the Results section into two parts. The new section "Synchronization on graphs and cell complexes" introduces the model and the notion of phase locking from a dynamical systems perspective, emphasizing intuition and physical interpretation. The subsequent section "Properties of cell complexes" then provides the necessary mathematical background.
- We now introduce phase-locked states first through explicit time series examples (Fig. 1 and Fig. 3), before giving the formal definition. This provides an intuitive entry point into the problem.
- We have added and expanded low-dimensional examples throughout the manuscript. In particular, the hexagonal ring is now worked out in detail as a guiding example, including explicit boundary operators, solution construction, and stability analysis.
- We have redesigned the key figures (Figs. 1 and 2) to better illustrate the main concepts and variables. In particular, we now show the boundary operators, interaction variables, and example solutions explicitly.
- We have introduced consistent terminology (e.g., "interaction variables") and added physical interpretations for key quantities such as $\boldsymbol{\theta}^{[\pm]}$, $\boldsymbol{\zeta}^{[\pm]}$, and the winding numbers.
- We have revised the presentation of the topological nonlinear Kirchhoff conditions by first explaining the conceptual idea and then providing the mathematical formulation.

suggestions:

1. give names to key critical variables, e.g., the B matrices, the angles $\theta^{[+]}$ and $\theta^{[-]}$,

Our reply: We thank the reviewer for this helpful suggestion and agree that introducing clear terminology for key variables improves readability.

Changes to the manuscript: We now explicitly introduce consistent names for the main quantities and explain them at their first occurrence:

- We introduce the boundary operators and their meaning: "The topology of a cell complex is encoded by boundary operators. The operator $\boldsymbol{B}_{[n]}$ encodes which $(n-1)$-cells lie in the boundary of each n-cell."
- We define and name the projected variables: "...the interaction variables

$$\boldsymbol{\theta}^{[-]} = \boldsymbol{B}_{[n]}\boldsymbol{\theta}, \qquad \boldsymbol{\theta}^{[+]} = \boldsymbol{B}_{[n+1]}^{\top}\boldsymbol{\theta}. \tag{7}$$

  The variable $\boldsymbol{\theta}^{[-]}$ measures phase differences across shared $(n-1)$-cell boundaries, while $\boldsymbol{\theta}^{[+]}$ measures phase accumulation around $(n+1)$-cells. For edge dynamics ($n=1$), these correspond to imbalances at nodes and circulations around faces, respectively."
- We also clarify the meaning of winding numbers and winding vectors at different levels of the presentation. We first introduce them in the context of the standard Kuramoto model via cycle sums of phase differences, then generalize this notion to higher-dimensional cycles of the cell complex, and finally provide the precise formulation in Eq. (23), where the vectors $\boldsymbol{z}^{[\pm]}$ represent winding numbers associated with independent cycles.

2. prepare a low dimentional example showing the most important steps, possibly to be included in a figure or in the supplementary information. (e.g., what are the B matrices in Figure 2? what are the decoupled equations (5) and (6) for the two examples in Figure 2?)

Our reply: We thank the reviewer for this helpful suggestion. We agree that explicit low-dimensional examples are essential to illustrate the main steps of the construction. In the revised manuscript, we have significantly expanded the presentation of such examples and made the relevant quantities explicit.

Changes to the manuscript:

- We have redesigned Fig. 1 (previously Fig. 2) to include two explicit low-dimensional examples, the hexagonal ring and the cube. For both, we now show the boundary operators $\boldsymbol{B}$ directly in the figure, together with sample time series illustrating phase-locked states.
- We have redesigned Fig. 2 (previously Fig. 1) to illustrate the construction of the topological nonlinear Kirchhoff conditions for a simple example (the tetrahedron), where the interaction variables $\psi_i^{[\pm]}$ are shown explicitly.
- We have expanded the analysis of the hexagonal ring to provide a complete worked-out example. In particular, we explicitly give the boundary operators and the corresponding $\boldsymbol{C}$ matrices, and demonstrate how the phase-locked states are obtained step by step.
- In addition, Fig. 3 now shows explicit time series for all three stable phase-locked states in the hexagonal ring, providing a direct illustration of the resulting dynamics.

Overall, the main contribution is Algorithm 1, which identifies all distinct phase-synchronized states in cell complexes and their stability. I am supportive of this result: I would appreciate (i) a discussion about number of equilibrium points and computational complexity, (ii) a discussion about the history and available software implementations of Algorithm 1 for standard Kuramoto models and Kuramoto models on simplicial complexes, and (iii) invariance under Abelian action (rotation of all angles by a constant angle) which is hidden in the current treatment.

Our reply: We thank the reviewer for this thoughtful and constructive comment. We agree that these aspects are important for understanding the scope and implications of Algorithm 1, and we have revised the manuscript accordingly.

Changes to the manuscript:

(i) **Number of equilibrium points and computational complexity.** We have added a discussion of the computational complexity of the algorithm and the number of equilibrium points. In particular, we now state: "A key aspect of this procedure is its computational complexity. In general, the procedure has a combinatorial component due to the choice of partitions $S^\bullet$ and S°, whose number scales exponentially as $2^{N_{[n]}}$. This can become prohibitive if one aims to enumerate all phase-locked states. In many applications, however, one is primarily interested in stable solutions. In this case, it is typically sufficient to restrict to partitions with $S^\circ = \emptyset$, which eliminates the combinatorial complexity associated with enumerating partitions." We further relate this to bounds on multistability in the subsequent section.

(ii) **Relation to existing approaches.** We have expanded the discussion of related work for the standard Kuramoto model. We now write: "While related approaches based on Kirchhoff-type formulations and cycle decompositions exist for the standard Kuramoto model on graphs [13–16], these are restricted to pairwise interactions. For the graph case, such methods can be used to derive necessary and sufficient conditions for the existence of phase-locked states in terms of cycle constraints [17, 18]. The present framework extends these ideas to higher-order networks, where multiple independent cycles across dimensions contribute to the solution space." This clarifies both the connection to and the distinction from existing methods.

(iii) **Invariance under global phase shifts.** We have clarified the invariance of the dynamics under global phase shifts (an Abelian symmetry) in the revised manuscript. In particular, we now state: "A subtle point is that the conditions $\frac{d}{dt}\boldsymbol{\theta}^{[\pm]} = \boldsymbol{0}$ do not fully fix the original variables $\boldsymbol{\theta}$. In particular, $\boldsymbol{\theta}$ may still contain a harmonic component that does not contribute to $\boldsymbol{\theta}^{[-]}$ or $\boldsymbol{\theta}^{[+]}$. This component affects the dynamics only in a trivial way, corresponding to a global or circulating shift of the phases that does not enter the interaction terms." This makes explicit that phase-locked states are defined modulo such symmetry transformations.

Beside my concerns about the clarity and accuracy of the presentation, I have to say that the existence of Algorithm 1 is relatively natural (given similar results for the standard Kuramoto) and I am slightly unsure about whether this result deserves to appear in Nature Communication.

Our reply: We thank the reviewer for this important comment. We agree that, once established, Algorithm 1 may appear conceptually natural, particularly in light of related results for the standard Kuramoto model on graphs. However, we would like to emphasize that the main contribution of our work is not the algorithmic structure itself, but its systematic extension to general cell complexes.

While Kirchhoff-type formulations and cycle-based approaches exist for graph-based Kuramoto models, their extension to higher-order structures is highly non-trivial. In cell complexes, phase-locked states are constrained simultaneously by lower- and higher-dimensional interactions, leading to coupled nonlinear conditions across multiple cycle spaces. To the best of our knowledge, no previous work provides a complete and constructive characterization of all phase-locked states in such settings.

Our results show that:

1. phase-locked states in cell complexes can still be decomposed into linear balance conditions and nonlinear topological constraints,
2. multiple independent winding numbers arise across dimensions, and
3. these lead to qualitatively new phenomena, including multistability cascades and universal boundary-size constraints.

In this sense, Algorithm 1 provides a unifying framework that renders these higher-order effects analytically tractable, rather than a straightforward extension of existing graph-based methods.

Changes to the manuscript: We have revised the manuscript to better highlight this conceptual advance and to more clearly position our results relative to the existing literature on the standard Kuramoto model. In particular:

- We now explicitly relate our approach to Kirchhoff-type formulations for graph-based Kuramoto models and recall the standard Kuramoto model before introducing the topological generalization.
- In Section "Synchronization on graphs and cell complexes" and Fig. 2 we first explain the nonlinear Kirchhoff conditions for the standard Kuramoto model before extending them to cell complexes.
- We clarify how the presence of multiple cycle spaces across dimensions fundamentally changes the structure of the solution space.
- We emphasize that the resulting multistability patterns have no direct analogue in pairwise interaction models.

---

[1] S. Barbarossa and S. Sardellitti, IEEE Transactions on Signal Processing **68**, 2992 (2020).
[2] J. Hoppe, V. P. Grande, and M. T. Schaub, arXiv preprint arXiv:2506.09726 (2025).
[3] A. E. Sizemore, C. Giusti, A. Kahn, J. M. Vettel, R. F. Betzel, and D. S. Bassett, Journal of Computational Neuroscience **44**, 115 (2018).
[4] T. M. Roddenberry, M. T. Schaub, and M. Hajij, in *IEEE International Conference on Acoustics, Speech and Signal Processing (ICASSP)* (2022) pp. 8852–8856.
[5] A. P. Millán, J. J. Torres, and G. Bianconi, Physical Review Letters **124**, 218301 (2020).
[6] M. Nurisso, A. Arnaudon, M. Lucas, R. L. Peach, P. Expert, F. Vaccarino, and G. Petri, Chaos **34**, 053118 (2024).
[7] D. Mulder and G. Bianconi, Journal of Statistical Physics **173**, 783 (2018).
[8] L. Giambagli, L. Calmon, R. Muolo, T. Carletti, and G. Bianconi, Physical Review E **106**, 064314 (2022).
[9] F. Battiston, G. Cencetti, I. Iacopini, V. Latora, M. Lucas, A. Patania, J.-G. Young, and G. Petri, Physics reports **874**, 1 (2020).
[10] C. Bick, E. Gross, H. A. Harrington, and M. T. Schaub, SIAM Review **65**, 686 (2023).
[11] P. S. Skardal and A. Arenas, Communications Physics **3**, 218 (2020).

[12] M. Lucas, G. Cencetti, and F. Battiston, Physical Review Research **2**, 033410 (2020).
[13] R. Delabays, T. Coletta, and P. Jacquod, Journal of Mathematical Physics **57**, 032701 (2016).
[14] D. Manik, M. Timme, and D. Witthaut, Chaos: An Interdisciplinary Journal of Nonlinear Science **27**, 083123 (2017).
[15] S. Jafarpour, E. Y. Huang, K. D. Smith, and F. Bullo, SIAM Review **64**, 59 (2022).
[16] A. Parameswaran, A. Benigni, D. Witthaut, and I. Bačić, arXiv preprint arXiv:2505.24818v2 (2025).
[17] C. Hartmann, P. C. Böttcher, D. Gross, and D. Witthaut, PRX energy **3**, 043004 (2024).
[18] I. Bačić, C. Hartmann, P. C. Böttcher, D. Gross, A. Benigni, and D. Witthaut, "Existence of phase-cohesive solutions of the lossless power-flow equations," TechRxiv Preprint DOI:10.36227/techrxiv.174611978.89382822/v1 (2025).

REPLIES TO THE REVIEWER COMMENTS.

We once thank the reviewers for their thorough reading of our work, and their positive feedback. In the following, we provide a detailed response to the reports. Original comments are given in black italic font, while our replies to the reviewers are given in blue (Our reply: sample comment).

REVIEWER #1

The authors did an excellent job of revising the paper. The new version is clearer, and the overall presentation has significantly improved. While some aspects still require refinement, this version is already of very good quality.

The Kirchhoff conditions are now intuitively clear, even though a few steps in the computation remain somewhat difficult to follow (see below). The examples are highly illustrative and effective. I particularly appreciated Figs. 3 and 4 along with their respective captions. Having read this revised version, I can now appreciate the beautiful theory developed by the authors.

Given that this is paper is considered for publication in a broad-audience journal, I believe the authors should endeavor to make their topic even more reader-friendly. They should avoid taking foundational concepts for granted when introducing topological signals and cell complexes. Moreover, a very brief overview of the dynamics of topological signals, beyond just the Topological Kuramoto model, would greatly benefit the community and familiarize more readers with this emerging field.

Our reply: We thank the reviewer for the careful final reading of our manuscript, for their positive assessment, and for the additional suggestions. We have implemented corresponding revisions throughout the manuscript, as detailed below.

Specific comments and suggestions are as follows:

At the beginning of Results, I think it would be best to write "networks" instead of "graph" in the title of the section. In fact, right in the first sentence the authors use "network".

Our reply: We have changed the section title accordingly and now use "networks" instead of "graphs".

In general, throughout the manuscript, both "graph" and "networks" are found. I'd suggest the use of one of the two, mentioning that they are equivalent at the beginning (given the community where this paper will circulate, I'd suggest the use of "network").

Our reply: We have adopted "network" as the preferred terminology throughout the manuscript. The only exception is when referring to the standard graph-theoretic object, "the graph (network) Laplacian".

After Eq. 3, I think it would be more correct to write "is the graph (network) Laplacian", rather than "is a graph Laplacian". If the authors write "a", it should be explained that this is the combinatorial Laplacian, not to be confused with the consensus nor the random walk one.

Our reply: We have revised the wording accordingly and now refer to it as "the graph (network) Laplacian."

Fig 1 is improved, but I still think it's not too clear, especially for scholars coming from networks and not too familiar with higher-order interactions. First of all, it's not clear that edges are not directed but it's just an orientation needed to define the boundary operators. Then, the faces are not well displayed. The cell-complexes both look like directed networks with some kind of vector field in the faces. I think this figure needs to be further improved (especially panels a and b).

Our reply: We have revised the visual design of Fig. 1**a,b**. In particular, we removed the arrows, added the variable labels $\theta_{[jk]}(t)$ on the edges to facilitate comparison with panels **e,f**, and highlighted the visible faces by color. We hope that these changes make the geometric structure and the role of orientations clearer.

It should be noted somewhere that, while the Laplacian is uniquely defined, the boundary operators are not and in principle other orientations would be possible.

Our reply: We have added the following clarification to the discussion of boundary operators: "While the Laplacian is uniquely defined, the boundary operators are not, in the sense that they depend on a chosen orientation."

Given the broad audience of this journal, some more explanations on L_{up} and L_{down} should be given.

Our reply: We have expanded the discussion of the up- and down-Laplacians and added the following explanation: "The superscripts "up" and "down" follow the standard terminology of Hodge Laplacians on cell complexes. The operator $\boldsymbol{L}^{[\mathrm{up}]}$ acts on $(n-1)$-cell variables and couples them through common incident n-cells; hence the coupling is mediated from the dimension above. Conversely, $\boldsymbol{L}^{[\mathrm{down}]}$ acts on $(n+1)$-cell variables and couples them through shared n-cell boundaries; hence the coupling is mediated from the dimension below."

I am not sure the dimensions of the cell-complexes are correct. If the cube has really n=3, then there should also be a volume-face, which, if I understand correctly, is not present. So n=2 for both cell complexes.

Our reply: We agree that the notation could be misleading in this context. We have therefore removed the references to $n = 2$ and $n = 3$ in the respective sentence.

When introducing n-cochains, rather than "vectors defined on n-cells", I think it clearer to write "state variables defined on n-cells". Also, an analogy with differential forms could help readers not familiar with this.

Our reply: We have revised the text accordingly. It now reads: "State variables, or more generally quantities, defined on n-cells are called n-cochains. They can be viewed as discrete analogues of differential n-forms: a 0-cochain assigns values to nodes, a 1-cochain to edges, a 2-cochain to faces, and so on."

When talking about the n-th Betti number, it should be specified that it is the number of n-dimension cycles (maybe also some examples could be given). In the present phrasing, it seems that the cycles can be of any dimension.

Our reply: When talking about the n-th Betti number, we now say: "...its dimension is given by the nth Betti number, i.e., the number of independent n-cycles (holes) in the complex." Given the space constraints, we have not added further examples at this point.

In Fig 2, it should be mentioned what are $z^{[+/-]}$ and possible given an explicit example. Also, an explicit mention of what is the winding number in the case of network should be given, as not every reader may be familiar with it.

Our reply: In Figure 2, we have added "with $z^{[\pm]} \in \mathbb{Z}$" next to the topological nonlinear Kirchhoff conditions. We refrain from adding a particular example here because the Figure explains the general principle. Explicit examples are provided in Fig. 1 and Fig. 3.

Regarding winding numbers, after Eq. (23), we have added the following: "As before, we have taken into account that phases are defined modulo 2π only. Hence, the right-hand sides do not necessarily vanish, but may take any value $2\pi\boldsymbol{z}^{[\pm]}$, where $z^{[\pm]}$ are vectors of integers of appropriate dimension which are referred to as generalized winding vectors."

The authors can now get an intuition on what the matrices C are, but still it's not completely clear to me how they can be obtained. Is there an exact relation with the corresponding boundary matrices which would satisfy the same condition?

Our reply: We now added a comment on obtaining the $\boldsymbol{C}$ matrices: "These matrices can be obtained directly by a standard nullspace computation, for example by Gaussian elimination, a rank-revealing QR decomposition, or a singular-value decomposition of the corresponding boundary matrices. Only their column span is relevant. Different choices of basis lead to equivalent parametrizations of the same solution space." Later, we also add "$\boldsymbol{C}_{[n+1]}$ and $\boldsymbol{C}_{[n]}^{\top}$ may be chosen from independent columns of the neighboring boundary matrices together with additional harmonic representatives whenever the corresponding homology or cohomology is nontrivial."

When obtaining the Kirchhoff conditions, I could follow up until before Eq. 19, then I got lost. Why is the general solution written as in Eq. 19?

Our reply: We have added additional explanation before Eq. (18), stating: "The form of the general solution follows from the elementary structure of the corresponding linear systems: solving Eqs. (15)-(16) for $\boldsymbol{K}_{[n+1]}\boldsymbol{\psi}^{[+]}$ and $\boldsymbol{K}_{[n]}\boldsymbol{\psi}^{[-]}$ gives one particular solution plus arbitrary components in the corresponding nullspaces. Transforming back to $\boldsymbol{\psi}^{[\pm]}$ yields..."

After Eq. 21, the authors say that the frequency vector can be used as a control parameter. But the frequency can be rescaled with the coupling strength, so it should be specified that the coupling strength needs to be fixed. Or, alternatively, that one of the two can be used as a control parameter.

Our reply: We modified the sentences on $\boldsymbol{\omega}$ as a control parameter to now say "For fixed coupling matrices $\boldsymbol{K}_{[n]}$ and $\boldsymbol{K}_{[n+1]}$, $\boldsymbol{\omega}$ acts as a control parameter: changing its entries shifts the affine space of candidate solutions and thereby determines which phase-locked states are admissible. Equivalently, in the case of homogeneous scalar coupling, the relevant control parameter is the ratio of natural frequencies and coupling strength."

The use of "symmetric" and "asymmetric" in dynamics on networks is tricky, because the reader might think that the support is somehow asymmetric. I suggest finding an alternative or specify, e.g., "permutation-symmetric", etc.

Our reply: We have adopted the term "permutation-symmetric" instead of "symmetric".

The fact that heterogeneous frequencies can turn unstable locked states into stable ones is very interesting. Is there a pairwise analogous of this?

Our reply: In response to this interesting observation, we expanded the discussion and added the following text together with the corresponding references:

"As ω_0 is increased, however, the frequency heterogeneity qualitatively changes the solution structure. In a narrow interval of ω_0, the value of the interaction variable $\theta_5^{[-]}$ of a stable solution crosses $\pi/2$ (Fig. 4**b**). Hence, the stable branch is no longer associated to the all-normal partition $S^\circ_{[0]} = \emptyset$ but by the mixed partition $S^\circ_{[0]} = [5, 6]$. Thus, heterogeneous intrinsic frequencies act as a control mechanism that selects which partition carries the stable phase-locked state. Increasing ω_0 further, a stable and an unstable collide and annihilate in a saddle-node bifurcation (Fig. 4**b**). Interestingly, both are associated to the branch $S^\circ_{[0]} = [5, 6]$. This is consistent with the broader picture in which loop flows around network cycles can stabilize phase-locked states with angle differences exceeding $\pi/2$ when interactions are pairwise [1, 2]. This phenomenon is closely related to the definiteness of generalized Laplacian matrices with negative weights [3]."

In the section discussing simplicial complexes, there's a typo when introducing Table I in the text ("I" is missing).

Our reply: We checked both the manuscript source and the generated PDF and could not reproduce the issue. The reference appears correctly in our version. Nevertheless, we carefully reviewed the relevant passage to ensure that the table citation is displayed correctly.

REVIEWER #2

I co-reviewed this manuscript with one of the reviewers who provided the listed reports. This is part of the Nature Communications initiative to facilitate training in peer review and to provide appropriate recognition for Early Career Researchers who co-review manuscripts.

Our reply: We thank the reviewer for their positive assessment of our revised manuscript and for their helpful comments during the review process.

REVIEWER #3

The authors have addressed all my concerns. I thank them for their clear responses and careful revision.

Our reply: We thank the reviewer for their positive assessment of our revised manuscript and for their helpful comments during the review process.

REVIEWER #4

The paper has been substantially reviewed. So much so that it appears to be a completely new document. I approve of the extensive changes. Each of my comments during the previous revision has been satisfactorily addressed and, even more, the revision is clearly compelling. The positive parts of the original submission has been strenghtened and, as such, I am glad to recommend acceptance.

Our reply: We thank the reviewer for their positive assessment of our revised manuscript and for their helpful comments during the review process.

FURTHER CHANGES TO THE MANUSCRIPT

During the final revision, we identified several additional references related to the counting of stable states in the pairwise Kuramoto model. We therefore added the following sentence after Eq. (28): "This recovers the known algebraic counting of stable twisted states on locally coupled Kuramoto rings [4, 5], whose multi-cycle generalization was given in [1]. An analogous bound holds for unidirectionally coupled rings [6]."

We have revised all figures according to Nature Communication's guidelines.

---

[1] R. Delabays, T. Coletta, and P. Jacquod, Journal of Mathematical Physics **58**, 032703 (2017).
[2] D. Manik, D. Witthaut, B. Schäfer, M. Matthiae, A. Sorge, M. Rohden, E. Katifori, and M. Timme, The European Physical Journal Special Topics **223**, 2527 (2014).
[3] D. Zelazo and M. Bürger, in *53rd IEEE Conference on Decision and Control* (IEEE, 2014) pp. 2895–2900.
[4] J. Ochab and P. F. Góra, Acta Physica Polonica B Proceedings Supplement **3**, 453 (2010).
[5] R. Delabays, T. Coletta, and P. Jacquod, Journal of Mathematical Physics **57**, 032701 (2016).
[6] J. A. Rogge and D. Aeyels, Journal of Physics A: Mathematical and General **37**, 11135 (2004).
